# The Role of Thailand in the International Trade in CITES-Listed Live Reptiles and Amphibians

**DOI:** 10.1371/journal.pone.0017825

**Published:** 2011-03-25

**Authors:** Vincent Nijman, Chris R. Shepherd

**Affiliations:** 1 Oxford Wildlife Trade Research Group, School of Social Sciences and Law, Oxford Brookes University, Oxford, United Kingdom; 2 TRAFFIC Southeast Asia, Petaling Jaya, Selangor, Malaysia; Smithsonian's National Zoological Park, United States of America

## Abstract

**Background:**

International wildlife trade is one of the leading threats to biodiversity conservation. The Convention on International Trade in Endangered Species of Wild Fauna and Flora (CITES) is the most important initiative to monitor and regulate the international trade of wildlife but its credibility is dependent on the quality of the trade data. We report on the performance of CITES reporting by focussing on the commercial trade in non-native reptiles and amphibians into Thailand as to illustrate trends, species composition and numbers of wild-caught vs. captive-bred specimens.

**Methodology/Principal Findings:**

Based on data in the WCMC-CITES trade database, we establish that a total of 75,594 individuals of 169 species of reptiles and amphibians (including 27 globally threatened species) were imported into Thailand in 1990–2007. The majority of individuals (59,895, 79%) were listed as captive-bred and a smaller number (15,699, 21%) as wild-caught. In the 1990s small numbers of individuals of a few species were imported into Thailand, but in 2003 both volumes and species diversity increased rapidly. The proportion of captive-bred animals differed greatly between years (from 0 to >80%). Wild-caught individuals were mainly sourced from African countries, and captive-bred individuals from Asian countries (including from non-CITES Parties). There were significant discrepancies between exports and imports. Thailand reports the import of >10,000 individuals (51 species) originating from Kazakhstan, but Kazakhstan reports no exports of these species. Similar discrepancies, involving smaller numbers (>100 individuals of 9 species), can be seen in the import of reptiles into Thailand via Macao.

**Conclusion/Significance:**

While there has been an increase in imports of amphibian and reptiles into Thailand, erratic patterns in proportions of captive-bred specimens and volumes suggests either capricious markets or errors in reporting. Large discrepancies with respect to origin point to misreporting or possible violations of the rules and intentions of CITES.

## Introduction

International wildlife trade is seen as one of the leading threats to biodiversity conservation [1]. It has been invoked as a vector for disease transmission to humans (including H5N1 spread by trade in birds [2] and SARS-associated coronavirus spread by trade in wild civets [3]) and wild animals (e.g. Chytridiomycosis spread by African clawed frogs [4]). International wildlife trade has also led to the introduction of invasive species, threatening individual species and ecosystems [5]. Recognizing the need to control this trade the Convention on International Trade in Endangered Species of Wild Flora and Fauna (CITES) has been ratified by 175 countries or states, at the time of writing. Globally CITES is the most important initiative to monitor and regulate the international trade of plants and animals, regulating trade of some 34,000 species, and reducing the threats associated with the over-harvesting of imperiled species for international trade. The credibility of CITES is dependant on the quality of the trade data as this informs decisions and garners political will and consensus among Parties [6], [7]. Recently, Phelps et al. [7] stressed the need for enhanced, rigorous analysis of existing trade data, as this would allow better decisions to be made on sustainable levels of trade (using Non-Detriment Findings), setting trade quotas and initiating suspensions.

One group of animals that are traded in large volumes (for skins, food and pets amongst others) are the amphibians and reptiles. With other factors, such as elimination of natural habitats, climate change and diseases, the collection of animals from the wild for commercial purposes has been invoked as a contributing factor to the decline, or even extinction, of individual species [8]–[10]. There have been some evaluations of the impact of commercial trade on certain taxa at a global level [11]–[13], with many of the studies having a more regional focus, such as the North American [14]–[16], European [17] or emerging markets [18], [19]. As noted [9] few countries record or make available data for species other than those regulated by CITES. Bickford et al. [20] argued that to increase the effectiveness of CITES and natural resource management a series of checks-and-balances and analysis of CITES data are needed, both for the traders and markets as well as for e.g. researchers and government officials.

In response to this call, here we focus the live trade of species of amphibians and reptiles into Thailand presumably largely to supply the exotic pet market (see methods). We assess the levels of trade in wild-caught and captive-bred individuals, and discuss the credibility of the captive-breeding claims. This work was motivated in part by emerging evidence that commercial captive-breeding of herpetofauna in certain countries is fraught with problems [21]–[23] and by the observation that increasingly exotic amphibians and reptiles are sold as pets in the Thai capital Bangkok [24]–[26].

Thailand became a Party to CITES in 1983 with the National Park, Wildlife and Plant Conservation Department being the lead CITES Management Authority –responsible for implementation and enforcement of the Convention- in the country. In the CITES National Legislation Project, Thailand has been rated in category 1, meaning that its legislation in believed to generally meet the requirements for the implementation of CITES. Selected species native to Thailand are protected under the Wild Animal Reservation and Protection Act B.E. 2535 (WARPA), which was last revised in 1992. All exotic species listed in the Appendices of CITES are also regulated by WARPA, under Chapter 4 – Importation, Exportation, Transitory movement of Wild Animals and Wild Animal check points. It specifically mentions, in Section 23, that “No person shall engage in the importation or exportation of wild animals [ ] listed in the prohibition list… [ ] unless these were obtained from breeding in captivity”. There is no mention of ‘possession’ or ‘domestic trade’ of species on the prohibition list, only exporting and importing, and Chapter 4 does not explicitly refer to exotic species.

## Results

A total of 75,594 individuals of at least 169 species of amphibians and reptiles were imported into Thailand in the period 1990–2007. The majority of individuals (59,895, 79%) were listed as captive-bred and a smaller number (15,699, 21%) as wild-caught. Chameleons and tortoises were traded in largest volumes, with frogs and snakes being traded in smaller numbers (Table 1).

**Table 1:** Main source countries for live captive-bred amphibians and reptiles imported into Thailand in the period 2003–2007.

Country	Frogs	Chameleons	Lizards	Snakes	Tortoises	Total	Period
Kazakhstan	2700 (16)	4078 (21)	700 (8)	0	2600 (6)	10078 (51)	2004–2006
Lebanon	0	148 (11)	0	0	788 (7)	936 (18)	2004
Indonesia	0	745 (5)	118 (3)	626 (1)	20 (1)	1509 (10)	2004–2007
Slovenia	0	0	153 (1)	200 (1)	1413 (1)	1766 (3)	2003–2006
Jordan	0	0	0	0	1001 (3)	1001 (3)	2005–2006
Zambia	0	0	0	0	3192 (2)	3192 (2)	2004–2007
Slovakia	0	2261 (1)	0	0	0	2261 (1)	2003–2004

Presented are total number of individuals with species number between brackets, countries are ordered by number of species.

**Figure 1:**
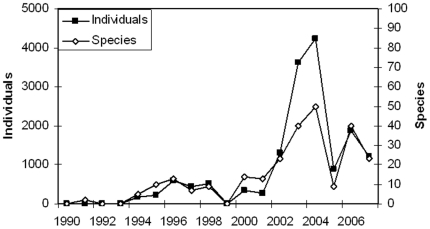
Import of wild-caught reptiles and amphibians. The figure shows the numbers of live wild-caught amphibians and reptiles imported into Thailand for the period 1990–2007.

Wild-caught individuals were imported into Thailand from 25 countries but the main trading partners are all African with Madagascar (8518 individuals, 33 species), Uganda (2350 individuals, 7 species), Tanzania (779 individuals, 13 species), Congo DRC (700 pancake tortoises *Malacochersus tornieri*), and Cameroon (465 individuals, 4 species) comprising the top five. In the 1990s relative small numbers of individuals of a few species were imported into Thailand, but in 2003 both volumes and species diversity increased rapidly for a few years only (Figure 1).

**Figure 2:**
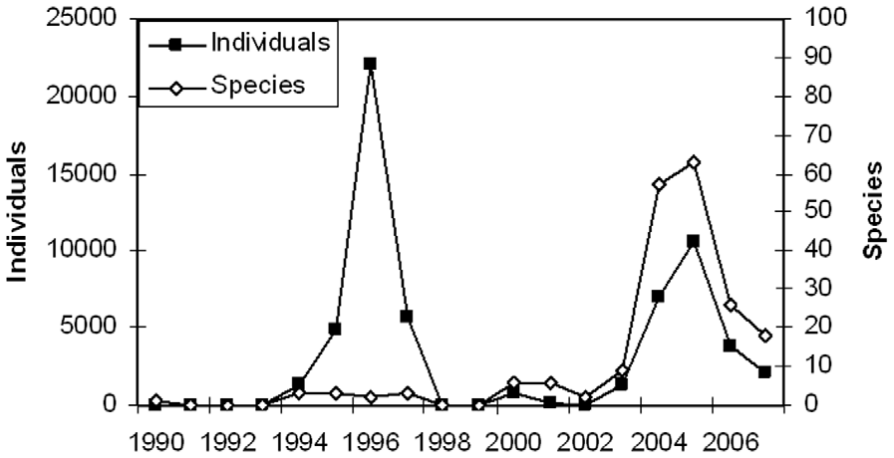
Import of captive-bred reptiles and amphibians. The figure shows the numbers of live captive-bred amphibians and reptiles imported into Thailand for the period 1990–2007, illustrating that from 2003 onwards both the number of individuals and the variety of species increased (note the different scale of the left y-axis when compared with Figure 1). The peak in the 1994–1997 is due to the import of large numbers of green iguana *Iguana iguana* from Colombia and El Salvador and spectacled caiman *Caiman crocodilus* from Venezuela.

Captive-bred individuals were imported from 41 countries. Volumes were in the low hundreds for most of the 1990s and early 2000s, with a major increase in numbers in 1994–1997 when large numbers of green iguana *Iguana iguana* from Colombia and El Salvador and spectacled caiman *Caiman crocodilus* from Venezuela were imported into Thailand (Figure 2). As with the import of wild amphibians and reptiles the number of species and number of individuals increased sharply from 2003 onwards. In some years more than 60 different species were imported. For this latter period, the main origin countries in terms of volume are Kazakhstan, Zambia, Slovenia and Indonesia with Lebanon, Kazakhstan and Indonesia exporting the largest number of species (Table 1). For six out of the seven countries that are the main suppliers for captive-bred individuals, when including re-exports, Thailand is a relative minor partner. Kazakhstan does not report any export of captive-bred amphibians or reptiles to any country, and for the period 2003–2007 in terms of volume, Thailand represents a mere 2 and 3% of the market for Jordan and Indonesia, respectively. For Slovenia (5%), Slovakia (5%) and Zambia (7%) these figures are slightly more significant, but only for Lebanon Thailand is the major trading partner with ∼40% of the total number of captive-bred reptiles and amphibians being exported to Thailand (the only other major importer of captive-bred reptiles and amphibians from Lebanon is Japan).

The import of live reptiles into Thailand via Macao is restricted to the year 2006 when 102 individuals of 9 species were re-exported from Macao. While Macao reported the re-export of these animals, for 78 individuals (76%) of 7 species there are no corresponding records of the animals ever being imported into Macao (Table 2).

**Table 2:** 2006 imports of live Appendix II reptiles into Thailand with Macao as re-exporter showing discrepancies in reporting

Species	origin	individuals	source	Imported into Macao
*Chamaeleo jacksonii*	Indonesia	12	C	not been reported as being imported/exported to Macao or China
*Calumma parsonii*	Kazakhstan	2	C	not been reported as being imported/exported to Macao or China
*Furcifer minor*	Kazakhstan	6	C	not been reported as being imported/exported to Macao or China
*Podocnemis unifilis*	Peru	12	F	not been reported as being imported/exported to Macao or China
*Chamaeleo dilepis*	Tanzania	6	W	not been reported as being imported/exported to Macao or China
*Chamaeleo rudis*	Tanzania	6	W	the species does not occur in the wild in Tanzania and has not been reported as being imported/exported into Macao or China
*Stigmochelys pardalis*	Zambia	24	C	12 individuals (origin Zambia) have been imported into Macao in 2006 with Thailand as re-exporter
*Testudo hermanni*	Slovenia	16	C	12 individuals (origin Slovenia) have been imported into Macao in 2006 with Thailand as re-exporter
*Furcifer pardalis*	Indonesia	18	C	24–36 individuals (origin Canada) have been imported into Macao in 2004–2005

[C = captive-bred, F = captive-born, W = wild-caught].

**Figure 3:**
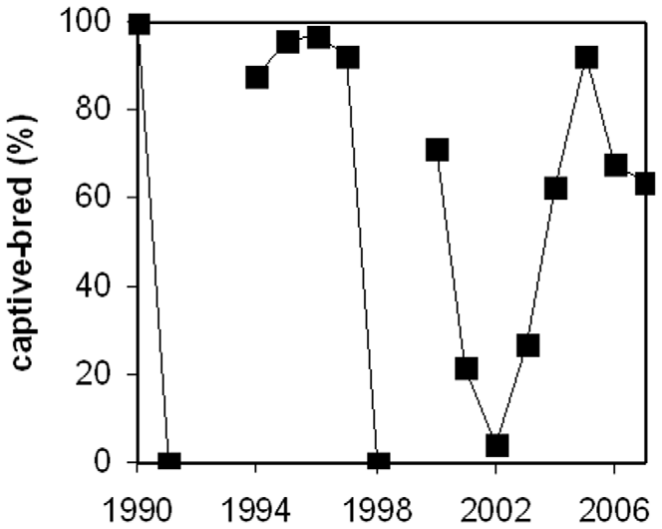
Captive-bred versus wild-caught animal imports. The figure shows the proportion of captive-bred amphibians and reptiles imported into Thailand. In some years most of the individuals are captive-bred, whereas in other years almost all are wild-caught (note that in 1992, 1993 and 1999 no trade in amphibians and reptiles is reported).

While the increase in captive-bred specimens may suggest a switch from wild-caught to captive-bred specimens there is no apparent pattern in the proportion of captive-bred amphibians and reptiles imported into Thailand. In some years 80% or more of the individuals are captive-bred, whereas in other years all are wild-caught, and this changes from one year to the next (Figure 3). While there appear to be no discrepancies between the source codes provided by the importing Party (i.e. Thailand) and the exporting Party (that is animals that are exported as ‘wild-caught’ are also imported as ‘wild-caught’ and animals that are exported as ‘captive-bred’ are also imported as ‘captive-bred’) there are large discrepancies in the volumes exported and imported, especially when it pertains to captive-bred specimens. For example, discrepancies in the amount imported and exported captive-bred specimens for Indian star tortoise *Geochelone elegans* total 1250 individuals, those for African spurred tortoise *G. sulcata* 1242 individuals, and those for leopard tortoise *Stigmochelys pardalis* 2024 individuals.

A total of 5441 individuals of 27 species listed as globally threatened were imported into Thailand in the period 1998–2007, with 1303 individuals of 6 species in the Critically Endangered category, 1129 individuals of 9 in the Endangered category and 3009 individuals of 12 species in the Vulnerable category (Table 3). Sixteen of the twenty-seven species that are currently considered globally threatened have been so for most of the ten-year period of the assessment, while eight species were assessed for the first time in 2008. In terms of the import of wild-caught specimens, most originated from Madagascar (1906 individuals of 7 species) and Mali (100 wild and 108 captive-bred African spurred tortoises). Captive-bred specimens mainly originated from Lebanon (2250 individuals from 11 species, note that this also includes re-exports from e.g. Kazakhstan), the United States (548 African spurred tortoises), Jordan (200 Mediterranean spur-thighed tortoises *Testudo graeca*) and Mali.

**Table 3:** Globally threatened CITES-listed amphibians and reptiles imported into Thailand from 1998–2007 highlighting the role of Madagascar in the export of wild-caught individuals and Lebanon in the export of captive-bred individuals.

IUCN status and species	Wild-caught	Source	Captive-bred	Source
**Critically Endangered (CR)**				
*Mantella aurantiaca* (2008; 1996 VU)	350	Madagascar		
*Mantella milotympanum* (2008)	37	Madagascar		
*Leucocephalon yuwonoi* (2000; 1996 DD)	4	Indonesia		
*Callagur [Batagur] borneoensis* (1996)	2	Malaysia		
*Pyxis arachnoides* (2008; 1996 VU)	10	South Africa	250	Lebanon
*Geochelone platynota* (1996)			650	Lebanon
**Endangered (EN)**				
*Mantella expectata* (2008)	385	Madagascar		
*Mantella viridis* (2008)	256	Madagascar		
*Mantella bernhardi* (2008)	100	Madagascar		
*Epipedobates tricolor* (2004)			100	Lebanon
*Phyllobates terribilis* (2004)			100	Lebanon
*Phyllobates vittatus* (2008)			100	Ukraine
*Cryptophyllobates [Hyloxalus] azureiventris* (2004)			40	Lebanon
*Indotestudo forstenii* (2000; 1996 VU)	28	Indonesia		
*Heosemys spinosa* (2000; 1996 VU)			20	Indonesia
**Vulnerable**				
*Mantella madagascariensis* (2008)	383	Madagascar		
*Mantella pulchra* (2008)	395	Madagascar		
*Furcifer campani* (1996)			220	Lebanon
*Furcifer labordi* (1996)			120	Lebanon
*Furcifer minor* (1996)			320	Lebanon
			6	Macau
*Cordylus giganteus* (1996)	10	South Africa		
*Phelsuma standingi* (1996)			100	Lebanon
*Osteolaemus tetraspis* (1996)			9	Denmark
*Kinixys homeana* (2006; 1996 DD)	20	Ghana		
*Geochelone sulcata* (1996)	100	Mali	108	Mali
			548	United States
			220	Lebanon
			20	Ghana
*Testudo graeca* (1996)			200	Jordan
*Malacochersus tornieri* (1996)			130	Lebanon
			100	Zambia

Between brackets is the year the species was first given its listed IUCN Red List status; if a previous assessment differed this is presented (DD = Data Deficient). Note that apart from Lebanon and Macau all countries listed are Party to CITES.

## Discussion

The reliability of the records in the CITES database is entirely dependent on the accuracy at which CITES Parties report these data. It has been well-documented that there are large discrepancies between officially reported import and export figures and the actual imports or export figures [13], [24], [27], [28]. Likewise, there may discrepancies between source codes, with switches between e.g. wild-caught and captive-bred, and unaccounted imports/exports. These inaccuracies, being deliberate or unintentional, undermine the credibility of CITES and lowers the confidence that allowable trade is biologically sustainable [7]. Recently, Smith et al. [29] reviewed current practises on assessing the impact of international trade on CITES-listed species and identified opportunities for scientific research. One of the ten key research areas they identified centred on developing case studies, such as the one presented here, with the aim of identifying and refining practical advice about making NDFs and creating awareness about effective NDF making practises. In addition they [29] highlighted the need for identifying discrepancies in the reporting of international trade.

Here we focussed on the trade in live amphibians and reptiles to supply the demand for the international pet trade into Thailand. Globally this trade involves millions of individuals annually [15], [17], [30] and in recent years Thailand has emerged as a significant importer of amphibians and reptiles, showing a clear increase in volumes imported. This includes substantial numbers of species that are globally threatened. The majority appears to involve captive-bred specimens. However, there is an erratic pattern of the ratio of captive-bred to wild-caught specimens being imported from one year to the next. Furthermore, there are major differences in the number of amphibians and reptiles that are reported as imported into Thailand. In some years not a single individual is import whereas in proceeding of following years 1000s of individuals are imported. This may point to a capricious market, with quick changing preferences for different species at the expense of others, or may refer to poor reporting.

While commercial captive breeding of amphibians and reptiles may relieve some pressure on wild populations, this is true only if the animals exported as captive-bred are indeed bred in controlled captive conditions out of parent stock that themselves were bred in similar conditions. The two countries that supply the bulk of the captive-bred specimens imported into Thailand are Kazakhstan and Lebanon [22]. Kazakhstan joined CITES in 2000 (hence it only has to report on trade in CITES listed species from 2000 onwards) but Lebanon is one of the few larger animal exporters not being a Party. Judging by the import into Thailand there appears to be significant captive-breeding facilities of amphibians and reptiles in Kazakhstan. Intriguingly, Kazakhstan itself does not report any export of amphibian or reptiles for the period, and all imports of captive-bred reptiles and amphibians from Kazakhstan are re-exported to Thailand via Lebanon (2004, 2005,) and Macao (2006). Given that Lebanon is not a Party it does not report to CITES and data on trade in CITES-listed species from Lebanon is only available from reported import data into CITES Parties, in this case Thailand. Macao is not a Party to CITES but it is a Special Administrative Region of China and China is a Party; Macao has a high degree of autonomy and maintains its own legal system, customs policy, and can send its own delegates to international organisations and events.

Concern about the import of ‘captive-bred’ *Testudo* spp. tortoises from Lebanon have been expressed [31] and with respect to the captive-breeding of Mediterranean spur-thighed tortoises the CITES Secretariat [32: 3] noted “Although captive breeding facilities are reported to exist in Lebanon, it is not clear whether they have the capacity to produce the number exported. The practice of rearing young from eggs laid by gravid wild females taken temporarily into captivity has been observed, although it is also not clear on what scale this takes place.” Similar concerns about the export of large numbers of allegedly captive-bred tortoises from Kazakhstan and Jordan to Japan, and to a lesser extent Thailand, have been indicated by Vinke and Vinke [23].

With respect to monitoring both legal and illegal trade it is important to realize that most wildlife trade routes pass through a limited number of trade hubs. These hubs do provide ample opportunities to maximize the effects of regulatory efforts as demonstrated with domestic animal trading systems (processing plants and wholesale and retail markets, for example). It is well-documented that there is a significant and open trade in exotic reptiles and amphibians in Thailand, especially at Chatuchak market in Bangkok, and this includes legally protected and Appendix I listed species [24], [26], [33]. In terms of wildlife trade, albeit not specifically reptiles and amphibians, the borders with neighbouring Myanmar, Laos and Cambodia can be porous, with several wildlife markets just across the border [7], [34]–[36]. Given Thailand's political status and long-term business relationships to industrial countries the county functions as a very important distributor to Southeast Asian and East Asian range states. With respect to exotic wildlife, Thailand functions as an important transit country for exotic species to especially Asian destinations [21] and the potential impact in the global trade with reptiles and amphibians can be significant. We agree with Stiles [37] that Thailand's leadership in the region with respect to biodiversity conservation brings incumbent responsibility to set a good example in controlling wildlife trade.

As to curtail the trade we recommend regular monitoring by Thai enforcement agencies and local and international NGOs of the markets in Thailand. Periodic surveys should be carried out, followed by detailed analysis to gauge the scale of trade and identify trends in species composition, countries of origin, and any end-market destinations beyond Thailand. If trade is deemed illicit, efficient measures to halt this trade should urgently be implemented. We urge the Thai authorities (Customs, Immigration, Quarantine and Security - CIQS) in the airports and other points of international entry and exit to be more vigilant to prevent large quantities of especially CITES I-listed species from being traded in Thailand. These authorities should ensure that their staff are regularly trained in CITES implementation and in other relevant fields, such as species identification and profiling [38]. We urge the CITES Management Authorities of Thailand, as well as the CITES Secretariat, to investigate the trade in wildlife from non-CITES Parties (Lebanon and Macao) to ensure it does not violate the regulations and intentions of CITES.

## Materials and Methods

We retrieved data on international trade from the WCMC-CITES trade database (http://www.unep-wcmc.org/citestrade) for the period 1990–2007. This database maintains all records of import and export of CITES-listed species as reported to the CITES Secretariat by Parties. We focus on commercial trade (listed with source-code ‘T’ in the database) in captive-bred (code ‘C’ and ‘D’) and wild-caught (‘W’) live reptiles and amphibians only, this being reported either by the importing country (Thailand) or exporting country. We excluded all non-commercial trade, e.g. exchange between zoos or export for scientific purposes. We assume that the vast majority of the amphibians and reptiles imported into Thailand are to supply the exotic pet market, but note that other than that the trade is ‘commercial’ no further data on purpose is provided in the WCMC-CITES trade database. For our definition of captive-bred we follow CITES where it refers to at least second generation offspring of parents bred in a controlled captive environment (or first generation offspring from a facility that is managed in a manner that has been demonstrated to be capable of reliably producing second-generation offspring in a controlled environment); it does not include specimens born in captivity to wild-caught parents and that are not considered as captive bred under CITES. [Note that all captive-breeding in this paper refers to captive-breeding in countries that export amphibians and reptiles to Thailand and not to specimens bred in Thailand]. Wild-caught refers to specimens that originate from the wild, and does not include individuals that are ranch-raised or progeny from gravid females captured from the wild.

Data on the conservation status was retrieved from the IUCN Red List website (www.iucnredlist.org) and we focussed on species that are Critically Endangered, Endangered or Vulnerable, excluding species that are listed as Near Threatened, Least Concern/conservation dependent or Data Deficient. Given that the conservation status of species changes over time we restrict our analysis of volumes and species compositions here to the last ten years for which data was available (i.e. 1998–2007).

An advanced draft version of this paper was send electronically and by postal mail (8 December 2009) to the Management Authorities of Thailand (CITES Office of the National Park, Wildlife and Plant Conservation Department and the Fisheries Resources Conservation Division of the Department of Fisheries) and Kazakhstan (Forestry and Hunting Committee and the Fishery Committee of the Ministry of Agriculture), using the addresses provided on the CITES website, for comments. We received a written response from the Director of the CITES Management Authority of Thailand on 5 January 2010, and we have taken his comments into account. No response was received from the Management Authorities from Kazakhstan.
